# Gelatin-based biomaterials as a delivery strategy for osteosarcoma treatment

**DOI:** 10.3389/fphar.2025.1537695

**Published:** 2025-01-28

**Authors:** Qifan Yang, Xingpeng Chen, Jing Liu, Yeteng He

**Affiliations:** ^1^ Department of Orthopedics, The First Hospital of Jilin University, Changchun, China; ^2^ Department of Orthopaedic Surgery, The First Affiliated Hospital of Shandong First Medical University & Shandong Provincial Qianfoshan Hospital, Jinan, China; ^3^ Department of Gynecology and Obstetrics, Jinan Maternity and Child Care Hospital Affiliated to Shandong First Medical University, Jinan, China

**Keywords:** osteosarcoma, gelatin, osteoblasts, osteoclasts, bone defects

## Abstract

Osteosarcoma is the most common primary malignant bone tumor. Although surgery and chemoradiotherapy have made some progress in the treatment of osteosarcoma. However, the high recurrence and metastasis rate of osteosarcoma and bone defects caused by surgery are still the main problems faced by osteosarcoma. Gelatin has excellent biocompatibility and biodegradability, and has made phased progress in tumor treatment. In the treatment of osteosarcoma, gelatin-based biomaterials can be used in delivery strategies to enhance the anti-tumor activity of osteosarcoma and can improve the appropriate compressive strength to improve the bone defects faced after surgery. At present, gelatin-based hydrogels, gelatin scaffolds, and gelatin-based nanoparticles have been reported in preclinical studies. In this article, we introduce the application of gelatin-based biomaterials in the treatment of osteosarcoma, and summarize and look forward to them.

## 1 Introduction

Osteosarcoma (OS) is the most common primary malignant bone tumor in children and adolescents (Beird et al., 2022). Osteosarcoma is more common in the distal femur, proximal tibia, and humerus, and osteosarcoma cells are derived from osteoblast mesenchymal stem cells (Dong et al., 2022). 4.4 patients with osteosarcoma per million population have been reported (Mirabello et al., 2009). Patients with osteosarcoma often present with excruciating, excruciating pain (Bian et al., 2023). Because osteosarcoma tends to occur in bone, its destruction of bone is usually prone to pathological fractures. OS occurs in osteoid produced by mesenchymal stem cells and osteoblast precursors or osteoblast precursors, so osteosarcoma is extremely malignant, and most of the OS will infiltrate into surrounding tissues and even metastasize at an early stage (Yang et al., 2020). The lung is the most common organ of osteosarcoma metastases (Jiang and He, 2023). Second, osteosarcoma cells have no specific markers, so early osteosarcoma is not easy to detect (Yuan P. et al., 2023). This is another reason for the high mortality rate of osteosarcoma.

Over the past few years, surgical resection and chemotherapy have made significant advances in the treatment of osteosarcoma (Ning et al., 2023). Surgical resection, combination chemotherapy, and targeted therapy for osteosarcoma have a 5 year survival rate of around 60%–70% for patients without metastases (Marina et al., 2004). The scope of surgical removal of osteosarcoma usually exceeds the ability of the bone to repair itself, which not only causes irreparable damage to the bone tissue, but also greatly reduces the patient’s desire to survive (Le Nail et al., 2018). The rationale for OS treatment has shifted from amputation to limb salvage to preserve local functional integrity and improve the patient’s quality of life (Simpson and Brown, 2018). The current standard chemotherapy drugs are mainly methotrexate, doxorubicin, ifosfamide and cisplatin (Zhang et al., 2018). However, as the disease progresses and osteosarcoma becomes resistant to chemotherapy drugs, survival rates for osteosarcoma have remained largely unchanged over the past few decades (Yuan B. et al., 2023). Osteosarcoma often brings great distress to clinicians and patients due to factors such as difficult treatment, recurrence and metastasis, and poor prognosis (Li S. et al., 2023). The toxic effects of chemotherapy drugs and the poor ability to target osteosarcoma cells often make chemotherapy not significantly progress in the treatment of osteosarcoma (Shi et al., 2023). Metastasis and recurrence of OS often result in a poor prognosis, so the 5 year survival rate for patients with OS who have metastases or recurrences is only 10%–20% (Marina et al., 2004). Therefore, the development of new and effective treatment strategies to improve patient survival is a top priority in the treatment of osteosarcoma.

**SCHEME 1:**
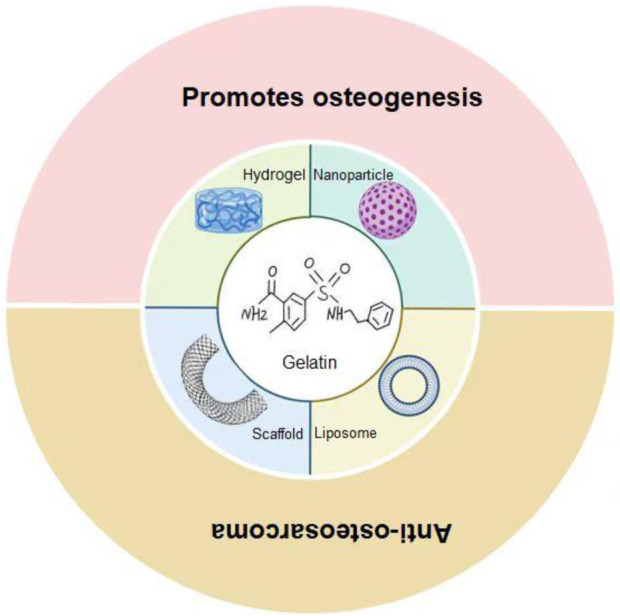
Gelatin-based biomaterials as a delivery strategy for osteosarcoma treatment. Gelatin-based hydrogels, scaffolds, and nanoparticles/liposomes have demonstrated diverse functions in the treatment of osteosarcoma. Hydrogels release active substances, deliver drugs, antimicrobial and promote bone regeneration; The stent can provide physical support, promote bone growth, and load drugs to inhibit the tumor; Nanoparticles/liposomes are unique in drug delivery and enhanced therapeutic efficacy.

In recent years, biomaterials have been widely used in various fields of nanomedicine and regenerative medicine due to their biological histocompatibility, targeting, and degradability (Xia et al., 2023; Xia et al., 2022a). In oncology treatment, biomaterials have shown strong potential trends in recent years (Xia et al., 2022b). As a type of biomaterial, gelatin is an attractive material for drug delivery and tissue engineering due to its excellent biocompatibility and biodegradability (Zhai et al., 2023). Gelatin-based biomaterials have been reported in the treatment of tumors (Danışman-Kalındemirtaş et al., 2022). However, gelatin-based hydrogels have weaker mechanical properties and are less immunogenic compared to collagen and its precursors (Maji et al., 2018). In physical and chemical modification, gelatin has adjustable mechanical strength and biological activity (Tan et al., 2023). Improved gelatin materials have been widely used in the treatment of osteosarcoma. Gelatin-based biomaterials can be used as a delivery strategy to enhance the anti-tumor activity of osteosarcoma (Scheme 1). In this article, we introduce the application of gelatin-based biomaterials in the treatment of osteosarcoma and summarize and look forward to them.

## 2 Gelatin properties

Gelatin is a denatured derivative of collagen containing a variety of functional amino acids, which has been widely used in tissue engineering due to its bioaffinity, excellent cell attachment, and low antigenicity (Su and Wang, 2015). Because it is derived from animal protein, it is non-toxic and degradable (Ahmed et al., 2020). Studies have shown that gelatin begins to denature above 40°C and loses its structural stability and hydrolyzes into different collagen fragments (Andreazza et al., 2023). What distinguishes gelatin from other natural peptides is the order in which amino acids are arranged in its branched chains, i.e., from the 17th amino acid residue, glycine (GLY) always occupies the third place (Hulmes et al., 1973). Therefore, after gelatin is degraded by proteases *in vivo*, it will increase cell adhesion and facilitate tissue regeneration (Echave et al., 2017). In addition, the porosity of gelatin ensures the diffusion of nutrients and oxygen, which promotes the normal growth of cells (Zhang et al., 2021). However, porosity is not directly proportional to cell survival. Studies have shown that irregular pores are shown to have lower cell viability (Seliktar, 2012). However, gelatin has poor mechanical stability, which limits its clinical application. Therefore, the combination of gelatin with other biomaterials to improve its stability is a commonly used improvement strategy (Lukin et al., 2022).

### 2.1 Hydrogel

Hydrogel is a hydrophilic macromolecular network, and hydrogel can fill various irregularly shaped defects for the treatment of osteosarcoma due to its softness (Li et al., 2019). The controllable and reversible swelling capacity of microgels with a diameter of 10 nm–100 μm can in turn improve drug delivery capabilities (Zhang et al., 2023). Reactive oxygen and nitrogen species (RONS) has recently been shown to have selective properties that kill cancer cells (Chauvin et al., 2017). Different tumor cells tolerate RONS differently, and studies have shown that injection of RONS around the tumor can promote osteosarcoma cell death (Van Boxem et al., 2017). Gelatin treated with cold atmospheric plasma is able to release RONS. Labay et al. gelatin treated by cold atmosphere plasma was used to deliver RONS to osteosarcoma (Labay et al., 2020). *In vitro* experiments confirmed that gelatin-released RONS could reduce osteosarcoma cell survival to 12%–23% after 72 h. In addition, gelatin can also inhibit the activity of MMP-2 and MMP-9 around tumors, thereby achieving anti-tumor effects (Hsu et al., 2019). Although gelatin has a certain anti-tumor effect, its effect is weak. Liu et al. prepared methacrylic acid gelatin/oxidized dextran/montmorillonite-strontium/polypyrrole (GOMP) hydrogels for the delivery of doxorubicin (DOX) (Liu et al., 2023b). The dual network structure of GOMP hydrogels formed by photoinitiator-initiated double bond polymerization and Schiff base reaction enables DOX to be payloaded and continuously released around the tumor. The compressive modulus of the GOMP@DOX hydrogel is 224.0 ± 40.2 kPa, which has good biocompatibility and biodegradability. In mice, it was confirmed that the GOMP@DOX hydrogel was fully degraded at 56 days. GOMP@DOX hydrogel can quickly release DOX within 12 h and reduce the toxic side effects of DOX. Compared with DOX, the hepatorenal toxicity of the GOMP@DOX hydrogel was reduced at 7 and 28 days in mice. In tumor-bearing mice, it was confirmed that GOMP@DOX hydrogel could effectively inhibit the proliferation and metastasis of osteosarcoma cells. Not only that, but GOMP@DOX hydrogels also provide mechanical support for bone regeneration. It not only improves the support capacity of the defective bone, but also provides the foundation for bone regeneration.

Giada Loi et al. developed a chitosan/gelatin hydrogel for the treatment of osteosarcoma using methylfuran group functionalization (Loi et al., 2023). Studies have shown that hydrogels can provide suitable compressive strength and crosslinking temperatures. After 14 days in the body, the hydrogel can be degraded into fragments, forming a negative interference with osteosarcoma cells. Not only that, the gelatin-based material will not cause necrosis of the surrounding tissues after degradation *in vivo*, indicating that its safety *in vivo* is satisfactory. Mojgan Ghanbari et al. developed a heat-sensitive injectable hydrogel that oxidizes alginate, gelatin, and carbon nitride quantum dots (CNQDs) (Ghanbari et al., 2021). Alginate hydrogels are a widely used biomaterial due to their biodegradability, biocompatibility, and ease of manufacturing (Balakrishnan et al., 2014). However, the poor adhesion of alginate limits its clinical application. Gelatin is combined with alginate to provide adhesion to alginate while increasing the rheological properties of the hydrogel. Not only that, the improved hydrogel enhances its mechanical strength, swelling rate, biodegradability and stability. Chen et al. used ALG/GelAGE to develop a hydrogel for the delivery of DOX and PDA particles (Chen et al., 2022). PDA can enhance the photothermal effect of hydrogels due to its excellent photothermal properties (Cheng et al., 2017). PDA particles have a diameter of about 200 nm and can be easily and uniformly dispersed in hydrogels compared to other photothermal agents. The hydrogel is porosity, and its porosity is 56.75% ± 0.64%. The DOX loading efficiency in PDA@DOX particles can be as high as 95%, which can fully release DOX to achieve anti-tumor effect. PDA@DOX particles can enhance the mechanical properties of hydrogels (compressive strength of 31.51 ± 0.84 KPa) and increase their stability. *In vivo* experiments confirmed that the modified hydrogel could promote apoptosis of tumor cells and reduce tumor volume in tumor-bearing mice.

After osteosarcoma resection, a large amount of bone is lost and cannot resist the attack of residual tumor cells and bacteria (Cha et al., 2023). Conventional bone implants do not have an antimicrobial effect and are not resistant to bacterial invasion (Ballatori and Hinds, 2016). Once an infection occurs, it poses severe pain and risk of amputation (Kadhim et al., 2022). Yin et al. developed an antimicrobial hydrogel using methacrylate gel (GelMA) and bioinert sulfonated polyetheretherketone (SP) (Yin et al., 2020). Upon the addition of tobramycin (TOB) to the hydrogel, the SP@MX-TOB/GelMA implant exhibits potent antimicrobial properties against gram-negative/gram-positive bacteria. Studies have shown that SP@MX-TOB/GelMA has good cytocompatibility and bone-promoting ability, and can also kill osteosarcoma cells and gram-negative/gram-positive bacteria. The addition of bone morphogenetic protein (BMP)-2 to the hydrogel can also enhance the osteogenic effect of the hydrogel (Yi et al., 2020).

Based on the premise that alginate can produce reactive oxygen species and nitrogen to enhance anti-tumor activity, Albert et al. prepared HA and alginate hydrogels for the treatment of osteosarcoma (Espona-Noguera et al., 2024). Hydrogels not only have the ability to promote cell proliferation or induce cell death, but also promote bone regeneration. Liu et al. fabricated a double-network hydrogel by photoinitiator-initiated double bond polymerization and Schiff base reaction (Liu et al., 2023a). This hydrogel is made of methacrylic acid gelatin/oxidized dextran/montmorillonite-strontium/polypyrrole (GOMP) as the main material. Polypyrrole can provide conductivity and high photothermal conversion ability for hydrogels under the premise of photothermal therapy, and the network structure of hydrogels can be used to deliver DOX to enhance its anti-tumor effect. The addition of strontium can safely achieve the ability of hydrogels to promote bone tissue regeneration.

### 2.2 Gelatin stents

Gelatin stents can be designed in different shapes to provide physical support at osteosarcoma defects, and their pores can facilitate bone ingrowth and accelerate bone repair (Moghaddam et al., 2024; Chrungoo et al., 2024). Chen et al. utilized a decellularized periosteal scaffold to load DOX-gelatin microspheres (Chen et al., 2019). The periosteal scaffold is the physicochemical removal of cellular components, showing excellent cytocompatibility and the ability to promote fibroblast adhesion and growth (Li et al., 2023b). DOX-gelatin microspheres can reduce the toxicity of DOX while maintaining its structural and functional stability. Local acellular scaffolds can locally deliver osteosarcoma chemotherapy drugs, reduce osteosarcoma cell proliferation and provide physical support for bone regeneration. To enhance bone regeneration, Adler et al. utilized silane-modified gelatin-polystyrene sulfonates to modify polydopamine (PDA) scaffolds to make PDA-coated stents (Adler et al., 2023). The PDA-coated scaffold has uniform porosity (122 ± 39 μm) that contributes to the formation of mineralized bone by osteoblast activity after implantation (Han et al., 2019). The compressive stress and Young’s modulus of PDA-coated stents were (0.7 ± 0.3 MPa) and (57 ± 24 Mpa), respectively, and they were stable in cancellous bone. PDA-coated scaffolds are stable and can be degraded by 15% in 4 weeks *in vivo*. In addition, the PDA-coated scaffold can enhance the properties of the hydroxyapatite layer *in vivo* and promote the proliferation and differentiation of osteoblasts. Ghorbani et al. modified PDA scaffolds using alginate and gelatin (Ghorbani et al., 2022). Compared with the PDA stent, the improved PDA stent was more stable (compressive strength of 0.69 ± 0.02 MPa). The surface of the PDA scaffold has a porous structure (1.37 ± 0.12 mm), which can be loaded with osteocalcin and osteomodulin, which can promote biomineralization and accelerate osteoinduced regeneration. In addition, the PDA-coated ADA-GEL scaffold supports osteosarcoma cell adhesion and proliferation.

Titanium is commonly used in orthopedic and dental implants due to its excellent mechanical properties, chemical resistance, and biocompatibility (Elias et al., 2015). Studies have shown that Ti scaffolds with pore sizes between 350 and 1,500 μm can achieve stiffness similar to that of human bone (Nune et al., 2018). However, titanium, as an implant, is not biologically active and lacks osseointegration capacity (Jiang et al., 2018). Cai et al. designed and developed the 3D-printed Ti6Al4V lattice scaffold with hydrothermally induced, photothermal conversion TiO2/TiP coating (HR-Ti) (Cai et al., 2021). The scaffold is also infused with gelatin/hydroxyapatite nanocomposites to provide the basis for cell adhesion/bone regeneration. The gelatin/hydroxyapatite nanocomposites have an average pore size of 7.02 nm, which provides good space for drug delivery. The pore size of the 3D-printed gel scaffold is 50–600 μm, and its porous structure facilitates the ingrowth of bone tissue. The compressive strength of the stent is 17.8 ± 1.6 Mpa, and the elastic modulus is 8.8 ± 0.2 GPa. Stent loaded drugs are released within 2 days with >90% release and 28 days for complete release. *In vitro* experiments have demonstrated that hydrogel scaffolds can promote osseointegration and anti-tumor effects at 12 weeks after surgery.

After surgical removal of osteosarcoma, postoperative bacterial infection triggers an inflammatory response and hinders the bone repair process. Therefore, local antimicrobial is also an important method for surgical resection of osteosarcoma. Huang et al. used N-acryloylglycanamide (NAGA)/methacrylic acid gelatin (Gel-MA) to make multifunctional hydrogels (GMNGs) (Huang et al., 2023). The combination of NAGA and Gel-MA endows GMNG with mechanical properties and controllable degradation ability, and GMNG has excellent superior tumor cell and antimicrobial ability. GMNG can effectively promote the formation of new bone in the body.

### 2.3 Nanoparticles/liposomes

Hydroxyapatite nanoparticles are widely used in orthopedic grafts due to their high surface-to-volume ratio, strong absorption capacity and excellent biocompatibility (Xia et al., 2022c). Liu et al. fabricated nanoparticles (DOX@nHAp NP) for DOX delivery using hydroxyapatite nanoparticles, gelatin, and polylactic acid fiber membranes (Liu et al., 2023b). DOX@nHAp NPs have an average size of 188.7 nm and have a 40% load efficiency for DOX. Polylactic acid membranes are able to give nanoparticles a stretching effect. DOX@nHAp NPs remain degraded *in vivo* for 20 days and continue to release DOX into the tumor microenvironment. DOX@nHAp NPs not only reduce the hepatorenal toxicity of DOX, but also promote osteoblast bone mineralization and enhance bone regeneration. Liao et al. developed a long-space nanoparticle for the treatment of osteosarcoma using gelatin methacrylate/chondroitin methacrylate, chondroitin sulfate, hydrogels, hybrid gold nanorods (GNRs) and nanohydroxyapatite (nHA) (Liao et al., 2021). Nanoparticles have good cytocompatibility and can enhance the photothermal therapy effect of osteosarcoma *in vivo*. Under light conditions, nanoparticles can penetrate tumor cell membranes faster without affecting surrounding tissues and cells. *In vivo* experiments have shown that nanoparticles can delay the growth of osteosarcoma cells and reduce their lung metastasis rate. The addition of nHA enhanced the bone mineralization capacity of the nanoparticles, which produced 65% more mineralized extracellular matrix (ECM) than the control group. Teotia et al. incorporated osteosarcoma cell-derived bone morphogenetic protein (BMP) and bisphosphonate (zoledronic acid) into gelatin cement to make gelatin biomaterials that promote cell proliferation and bone regeneration (Teotia et al., 2016). The particle size of gelatin cement is less than 40 μm, and the compressive strength is 6.9 ± 0.5 Mpa. Around tumors, the release of BMP from gelatin cement promotes bone regeneration. *In vivo*, gelatin cement promoted osteoblast adhesion on day 28 and detected Runt-related transcription factor-2 (RUNX2); collagen type I (COLI); bone sialoprotein (BSP); osteocalcin (OCN); Expression of glyceraldehyde-3-phosphate dehydrogenase (GAPDH). Within 16 weeks, it can promote trabecular bone regeneration.

The rapid degradation properties of gelatin are enhanced by the formation of gelatin modified by photo- or chemically induced polymerization of methacrylates (Yue et al., 2015). GelMA is widely used in biomedicine due to its biodegradability and variable physical properties (Xin et al., 2017). Wu et al. used GelMA to make nanoliposomes for the treatment of osteosarcoma (Wu et al., 2018). The average diameter of GelMA nanoliposomes is 119.6 ± 2.3 nm, and the compressive strength is 30.82 ± 1.49 N. Studies have shown that GelMA nanoliposomes are fully degraded *in vivo* for 21 days, and gemcitabine can be released (94.97% ± 2.20%) after 100 h *in vivo*. In an *in vivo* tumor-bearing mouse model, the growth of GelMA nanoliposome-treated mouse tumors at 14 days was significantly lower than that of the control group. Although this experiment did not verify whether GelMA nanoliposomes have sufficient compressive strength and support around osteosarcoma. However, in another study, the 3D-printed GelMA hydrogel can provide a solid foundation for bone regeneration, and the addition of angiogenesis factor and matrix metalloproteinase to the hydrogel can prevent mechanistic signaling and promote angiogenesis (Lin et al., 2023). However, it is important to note that an elevated MMP2/MMP9 activity ratio is associated with a poor response to chemotherapy in osteosarcoma (Kunz et al., 2016).

Nanoparticles alone cannot achieve on-demand and local delivery strategies (Patenaude and Hoare, 2012). Jalili et al. utilized GelMA, magnetic nanoparticles (MNPs), and poly (N-isopropylacrylamide-co-acrylamide) (poly (NIPAM-co-AM)) to make heat-responsive nanohydrogels for DOX delivery (Jalili et al., 2017). The loading rate of 150 nm of magnetic nanoparticle nanoparticles to DOX is 42.1% ± 8.1%. *In vivo* experiments have shown that within 24 h, magnetic nanoparticles can release 75.1% of DOX for chemotherapy for osteosarcoma. Within 48 h, tumor cell death can be significantly caused. Lin et al. synthesized GelMA for carrying photoinitiators to enhance antitumor activity (Lin et al., 2016). Studies have shown that glutathione can scavenge oxidative free radicals and protect cells in a viable state (Monostori et al., 2009). Albert et al. developed alginate-based hydrogels for the enhancement of peritumoral plasma-derived ROS and reactive nitrogen (Espona-Noguera et al., 2024). Low levels of reactive oxygen species and reactive nitrogen stimulate cell proliferation, while high concentrations of reactive oxygen species and reactive nitrogen are harmful (Privat-Maldonado et al., 2019). High doses of reactive oxygen species and reactive nitrogen have been shown to trigger cell death, endogenous RONS production, DNA damage, and lipid peroxidation in cancer cells (Malyavko et al., 2020). Alginate hydrogel solves the shortcomings of rapid and poorly controlled delivery of reactive oxygen species and reactive nitrogen that are easily diluted by body fluids, and can release a large amount of reactive oxygen species and reactive nitrogen in the local tumor. In tumor-bearing mice for 3 weeks, it was found that alginate hydrogel could greatly reduce tumor volume.

## 3 Conclusion

Osteosarcoma seriously affects the quality of life of adolescents. At present, there is no good clinical way to deal with the metastasis of osteosarcoma and the complications caused by surgery. Biomaterials have developed rapidly in tissue engineering and regenerative medicine in recent years, and have gradually become a novel modality in tumor treatment. As one of the important members of biomaterials, gelatin has good biocompatibility and has made phased progress in tumor treatment. The properties of gelatin make it an ideal biomaterial base, and the hydrogels, scaffolds, and nanoparticles/liposomes developed based on them have demonstrated a variety of functions in the treatment of osteosarcoma. Hydrogels release active substances, deliver drugs, antimicrobial and promote bone regeneration; The stent can provide physical support, promote bone growth, and load drugs to inhibit the tumor; Nanoparticles/liposomes are unique in drug delivery and enhanced therapeutic efficacy.

At present, its clinical application is rarely reported, but preclinical studies have demonstrated its potential. In the future, it is necessary to deeply explore the mechanism of action *in vivo*, compare and analyze different materials to optimize selection, strengthen standardized production and quality control, carry out multi-center clinical studies to accumulate data, and explore combination with existing therapies and personalized treatment strategies, and comprehensively consider cost-effectiveness. With the deepening of research, gelatin-based biomaterials are expected to break through the existing dilemma, bring a new dawn for the treatment of osteosarcoma, and significantly improve the prognosis and quality of life of patients.
